# Antiphospholipid Syndrome and Acute HIV Infection

**DOI:** 10.3201/eid1602.090728

**Published:** 2010-02

**Authors:** Jaime Solís Díaz, Juan Gómez Octavio, Manuel L. Fernández Guerrero

**Affiliations:** Universidad Autónoma de Madrid, Madrid, Spain

**Keywords:** Antiphospholipid syndrome, HIV infection, acute retroviral syndrome, HIV/AIDS and other retroviruses, viruses, letter

**To the Editor:** Patients with acute HIV infection frequently experience a syndrome characterized by fever, sore throat, lymphadenopathy, maculopapular rash, and lymphomonocytosis, which mimics acute infectious mononucleosis, 3–6 weeks after primary infection (*1*). Aseptic meningitis, encephalitis, and peripheral neuropathy are the most commonly observed features. In contrast, antiphospholipid syndrome complicated with pulmonary emboli is not commonly associated with acute retroviral syndrome. The following case should prompt clinicians to consider an expanded clinical scope of initial signs and symptoms for acute HIV infection.

A 28-year-old homosexual man was admitted to a hospital in Madrid, Spain, on June 22, 2009, with fever, pharyingitis, and myalgias. Generalized lymphadenopathy was found on examination. Lymphomonocytosis and mild elevation of serum aspartate aminotransferase and serum alanine aminotransferase levels were found. Chest radiographs showed no abnormalities. Results of a commercial ELISA for HIV-1 and HIV-2 were negative. Results of a p24 antigen-capture assay were positive, and viral load measured by reverse transcription–PCR (RT-PCR, Amplicor; Roche Molecular Diagnostics, Pleasanton, CA, USA) was 2,600,000 copies RNA HIV/mL. CD4+ T-cell count was 297 cells/μL.

The patient was discharged with instructions to take acetaminophen, but he was readmitted 1 week later with recurring fever, pleuritic chest pain, and shortness of breath. He was febrile (38.5ºC), tachycardic, and tachypneic and had a blood pressure of 155/72 mm Hg and generalized lymphadenopathy. Blood tests showed a hemoglobin level of 10.6 g/dL, leukocyte count of 5,160 cells/μL, and thrombocyte count of 293 cells/μL. Results of renal function tests were within normal limits as were serum aminotransferase levels. Lactate dehydrogenase level was 698 IU/L (reference range 211–423 IU/L) and d-dimer was 3,414 μg/L (reference range 68–494 IU/L). Fibrinogen levels, prothrombin time, and partial thromboplastin time were normal. Chest radiographs showed a small area of pleural effusion on the left side. A computed tomographic scan of the chest showed multiple pulmonary emboli with areas of parenchymal infarction.

Antibodies against phospholipids (PLs) and β_2_-glycoprotein I (β_2_GPI) measured by ELISA were detected at high titers: immunoglobulin (Ig) M anticardiolipin + 72 U MPL/mL (positive at >20 U MPL/mL), IgG anticardiolipin + 158 U GLP**/**mL (positive at >20 U GLP/mL), IgG anti-β_2_GPI + 210 U/mL (positive at >10). Results of screening tests for thrombophilia and other autoantibodies were within normal limits.

The patient was treated with low molecular weight heparin, oxygen, and analgesics. His fever subsided, and he was discharged a few days later while continuing to receive acenocoumarol, an oral coumarin anticoagulant. Results of a repeated HIV ELISA were then positive. Western blot assay confirmed the presence of antibodies to p24, gp41, and gp120/160.

One month after discharge, the patient was doing well. Titers of PL antibodies had declined (IgM anticardiolipin, negative; IgG anticardiolipin, 54; IgG antibody against β_2_GPI 90). Viral load was 762,000 copies of HIV-1 RNA/mL, and CD4+ T-cell count was 320 cells/μL. At follow-up, 2 months after symptom onset, he was asymptomatic, and PL antibody titers continued to decline; antibodies against β_2_GPI were undetectable, and only IgG anticardiolipin was still detected at lower titers (+33). Viral load was 129,000 copies/mL, and CD4+ lymphopenia was slowly improving (408 cells/μL). He was receiving anticoagulant therapy but not antiretroviral drugs.

Antibodies against PLs have been commonly found in patients with autoimmune diseases such as systemic lupus erythematosus and primary antiphospholipid syndrome, in which clinical manifestations (mainly thrombotic events) have been directly attributed to antibodies against PLs. In these patients, antibodies against PLs are specific for a neoepitope constituted by the union of β_2_GPI, a lipid-binding coagulation inhibitor, to the cellular membrane phospholipids (*2*). In addition, these antibodies have been observed in some acute viral and bacterial infections as a manifestation of the intense antigenic stimulation of the immune system. These antibodies recognize lipid components of cellular membrane and have no direct role in the coagulation pathway, and their presence probably reflects intense antigenic stimulation of the immune system. Because of the lack of a statistical association between these antibodies and development of thrombotic events, the presence of these antibodies is thought to be an epiphenomenon and of no clinical relevance (*3*,*4*).

Anticardiolipin antibodies and, less frequently, β_2_GPI antibodies also have been found in patients with chronic HIV infection, but their association with thrombotic events has not been proven (*5*). However, cases of antiphospholipid syndrome in HIV-infected patients have been anecdotally reported, prompting clinicians to reconsider the real role of these antibodies, particularly B_2_GPI antibodies, which are thought to be more specific for antiphospholipid syndrome. Avascular bone and cutaneous necrosis and deep vein thrombosis and pulmonary emboli were the most common manifestations of antiphospholipid syndrome (*6*–*8*).

In HIV-infected patients, PL antibodies and β_2_GPI antibodies have been strongly linked with level of viral replication (*9*). In our patient, the levels of viral load and PL antibodies seemed to run in parallel, with high concentrations of both at hospital admission and simultaneous decline over time. This observation suggests that patients with high levels of viremia, such as those with acute retroviral infection, could be at risk for high titers of PL antibodies and thrombotic events. Testing for antibodies in these patients should be considered as part of routine examination.
